# Febrile palpebral edema

**DOI:** 10.1016/j.jdcr.2021.06.015

**Published:** 2021-06-14

**Authors:** Mohammad Yassine Chérif, Bertrand Richert

**Affiliations:** a Internal Medicine Department, Brugmann University Hospital, Université Libre de Bruxelles, Brussels, Belgium; b Department of Dermatology, Brugmann University Hospital, Université Libre de Bruxelles, Brussels, Belgium

**Keywords:** EBV, Hoagland sign, infectious mononucleosis, palpebral edema, AD, atopic dermatitis, EA, early antigen, EBV, Epstein-Barr virus, EBNA, Epstein-Barr virus nuclear antigen, IgM, immunoglobulin M, VCA, viral capsid antigen

A 2-year-old patient presented to the emergency ward with a 3-day history of fever, sore throat, fatigue, and loss of appetite. Personal and familial past medical history were unremarkable. Physical examination revealed a calm and febrile child with slight conjunctival hyperemia, bilateral upper palpebral edema (Fig 1), bilateral nonexudative tonsillitis, and cervical lymphadenopathy. Skin examination was normal. White blood cell count showed a significant lymphocytosis with a majority of atypical lymphocytes (Fig 2). Other significant laboratory results were an elevated C-reactive protein level and mild transaminitis. Blood and urine cultures were negative.

**Fig 1:**
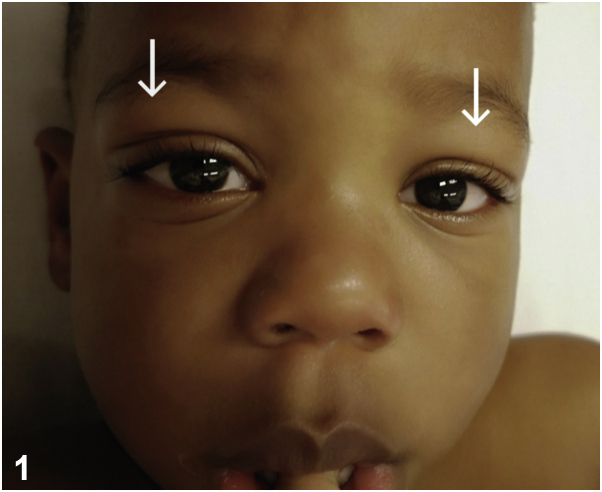

**Fig 2:**
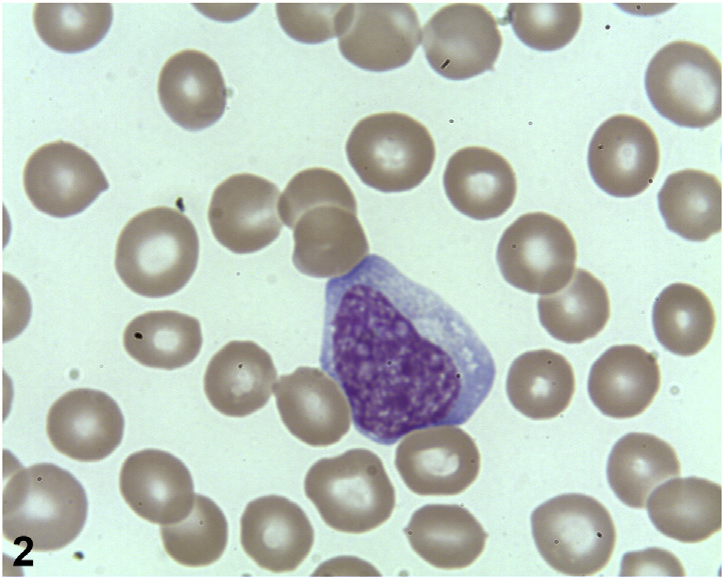

**Question 1: What is the most likely diagnosis?**

A. Atopic dermatitis (AD) flareup
B. Kawasaki disease
C. Trichinosis
D. Herpetic blepharitis
E. Infectious mononucleosis

**Answers:**

A. Atopic dermatitis (AD) flareup – Incorrect. Although a flareup of AD may accompany viral infection, there is no erythema, pruritus, or scaling of the eyelids. Integument examination did not show any symptoms of AD. Dennie-Morgan infraorbital folds are a double crease on the lower eyelids and are a minor criterion of AD. This sign was not observed in the patient.
B. Kawasaki disease – Incorrect. Kawasaki disease is a systemic vasculitis that occurs mainly in children. It combines persistent high fever for at least 5 days and 4 of the 5 following criteria: bilateral conjunctival congestion, lips and oral cavity alterations (cheilitis, strawberry tongue, and polymorphous rash), changes of the extremities, and cervical adenopathy without any other explanation.^1^ There is no specific testing. Main complication is coronary aneurysms. Echocardiography is a must.
C. Trichinosis – Incorrect. Trichinosis is a parasitic infection caused by nematodes, and it occurs worldwide secondary to the consumption of undercooked meat. Although palpebral edema is described in the larval dissemination phase, it typically manifests as a triad of symptoms, including fever, myalgia, and edema (particularly palpebral).^2^ Blood tests may reveal an elevation of creatine kinase and eosinophilia.
D. Herpetic blepharitis – Incorrect. Herpetic blepharitis is typically unilateral and manifests as monopalpebral vesicular rash, eyelid edema, skin erythema, and acute local pain.^2^ Atypical lymphocytosis is extremely unusual.^3^
E. Infectious mononucleosis – Correct. Infectious mononucleosis is caused by Epstein-Barr virus (EBV) and usually presents with a triad of fever, pharyngitis, and lymphadenopathy.^4^ A transient edema of the bilateral upper eyelids, known as Hoagland sign, is an occasional early sign of the infection. This finding is more commonly reported in pediatric patients compared with adults.^5^ The pathophysiology remains unclear; palpebral edema may be related to the lymphatic obstruction or lymphocyte infiltration of the lachrymal glands by lymphoproliferation.^5^

**Question 2: Which one of the following tests is expected to be found with the diagnosis of the presented case?**

A. Negative heterophile antibodies
B. Immunoglobulin M (IgM)-viral capsid antigen (VCA)^−^
  IgG-VCA^+^
  anti-Epstein-Barr virus nuclear antigen (EBNA)^−^
  antiearly antigen (anti-EA)^−^
C. IgM-VCA^+^
  IgG-VCA^+^
  anti-EBNA^−^
  anti-EA^+^
D. IgM-VCA^−^
  IgG-VCA^−^
  anti-EBNA^−^
  anti-EA^−^
E. IgM-VCA^−^
  IgG-VCA^+^
  anti-EBNA^+^
  anti-EA^−^

**Answers:**

A. Negative heterophile antibodies – Incorrect. Heterophile antibodies may be sufficient in case of positivity with a typical clinical picture of EBV infection. Otherwise, anti-VCA and anti-EBNA are required for the diagnosis. Moreover, young children aged <4 years may not develop a positive heterophile antibody response during EBV primoinfection.^4^
B. Immunoglobulin M (IgM)-viral capsid antigen (VCA)^−^
  IgG-VCA^+^
  anti-Epstein-Barr virus nuclear antigen (EBNA)^−^
  antiearly antigen (anti-EA)^−^ – Incorrect. This serologic profile is consistent with a convalescent infection.
C. IgM-VCA^+^
  IgG-VCA^+^
  anti-EBNA^−^
  anti-EA^+^ – Correct. This serologic profile is consistent with a EBV primoinfection. Anti-EA antibodies are the markers of acute infection, appearing in early disease.^4^ IgM anti-VCA are also usually found at the onset of the disease but studies report the existence of false positive results, especially with cytomegalovirus infections.^4^
D. IgM-VCA^−^
  IgG-VCA^−^
  anti-EBNA^−^
  anti-EA^−^ – Incorrect. This serologic profile is not consistent with an EBV infection.
E. IgM-VCA^−^
  IgG-VCA^+^
  anti-EBNA^+^
  anti-EA^−^ – Incorrect. This serologic profile is consistent with a remote past infection. IgG anti-VCA may be either negative or positive during the early infection but shows life-long persistance. On the other hand, anti-EBNA antibodies usually appear weeks after the infection onset. The presence of IgG anti-VCA and anti-EBNA in acute illness rules out a primary EBV infection.^4^

**Question 3: Which one of the following is not an EBV-associated disease?**

A. Kaposi sarcoma
B. Nasopharyngeal carcinoma
C. Gastric carcinoma
D. Hodgkin lymphoma
E. Burkitt lymphoma

**Answers:**

A. Kaposi sarcoma – Correct. Kaposi sarcoma is associated with human herpesvirus 8.
B. Nasopharyngeal carcinoma – Incorrect. In endemic regions, nasopharyngeal carcinomas are virtually all positive for EBV DNA.^4^
C. Gastric carcinoma – Incorrect. Gastric carcinomas that are associated with EBV represent approximately 10% of this type of cancer.^4^
D. Hodgkin lymphoma – Incorrect. Hodgkin lymphoma is a well-known EBV-associated disease, especially in underdeveloped countries.^4^
E. Burkitt lymphoma – Incorrect. Almost all Burkitt lymphomas have an EBV-associated etiology.^4^

## Conflicts of interest

None disclosed.
